# The CIVISANO protocol: a mixed-method study about the role of objective and perceived environmental factors on physical activity and eating behavior among socioeconomically disadvantaged adults

**DOI:** 10.1186/s13690-022-00956-6

**Published:** 2022-10-05

**Authors:** Suzannah D’Hooghe, Yasemin Inaç, Eva De Clercq, Benedicte Deforche, Sarah Dury, Stefanie Vandevijvere, Nico Van de Weghe, Delfien Van Dyck, Karin De Ridder

**Affiliations:** 1 Sciensano, Department of Epidemiology and Public Health, Brussels, Belgium; 2 Ghent University, Faculty of Medicine and Health Sciences, Department of Public Health and Primary Care, Ghent, Belgium; 3 Vrije Universiteit Brussel (VUB), Faculty of Psychology and Educational Sciences, Adult Educational Sciences, Brussels, Belgium; 4 Ghent University, Faculty of Sciences, Department of Geography, Ghent, Belgium; 5 Sciensano, Department of Chemical and Physical Health Risks, Brussels, Belgium; 6 Vrije Universiteit Brussel (VUB), Faculty of Physical Education and Physiotherapy, Department of Movement and Sport Sciences, Brussels, Belgium; 7 Ghent University, Faculty of Medicine and Health Sciences, Department of Movement and Sports Sciences, Brussels, Belgium

**Keywords:** Obesity, Socioeconomic status, Diet, Physical activity, Community-based participatory research, Geographic information systems

## Abstract

**Background:**

Overweight and obesity have a strong socioeconomic profile. Unhealthy behaviors like insufficient physical activity and an unbalanced diet, which are causal factors of overweight and obesity, tend to be more pronounced in socioeconomically disadvantaged groups in high income countries. The CIVISANO project aims to identify objective and perceived environmental factors among different socioeconomic population groups that impede or facilitate physical activity and healthy eating behavior in the local context of two peri-urban Flemish municipalities in Belgium. We also aim to identify and discuss possible local interventions and evaluate the participatory processes of the project.

**Methods:**

This study (2020–2023) will use community-based participatory tools, involving collaborative partnerships with civic and stakeholder members of the community and regular exchanges among all partners to bridge knowledge development and health promotion for socioeconomically disadvantaged citizens. Furthermore, a mixed-methods approach will be used. A population survey and geographic analysis will explore potential associations between the physical activity and eating behaviors of socioeconomically disadvantaged adults (25–65 years old) and both their perceived and objective physical, food and social environments. Profound perceptive context information will be gathered from socioeconomically disadvantaged adults by using participatory methods like photovoice, walk-along, individual map creation and group model building. An evaluation of the participatory process will be conducted simultaneously.

**Discussion:**

The CIVISANO project will identify factors in the local environment that might provoke inequities in adopting a healthy lifestyle. The combination of perceived and objective measures using validated strategies will provide a robust assessment of the municipality environment. Through this analysis, the project will investigate to what extent community engagement can be a useful strategy to reduce health inequities. The strong knowledge exchange and capacity-building in a local setting is expected to contribute to our understanding of how to maximize research impact in this field and generate evidence about potential linkages between a health enhancing lifestyle among socioeconomically disadvantaged groups and their physical, food and social environments.

## Background

An important determinant of overweight and obesity is a low socioeconomic status (SES). Overweight and obesity disproportionally affects individuals with lower SES, putting them at higher risk for chronic diseases (such as cardiovascular diseases, type II diabetes, cancer and depression) and thus, contributing to growing health inequities [1, 2, 3]. Furthermore, low SES is related to lower levels of physical activity and more unhealthy eating behaviors which lead to the development of overweight and obesity [4, 5, 6, 7].

Socioeconomic inequities in physical activity and diet cannot be explained exclusively by poorer accessibility and availability of physical activity facilities or healthy food options, lack of social support, financial constraints to afford healthy food or pay fees (e.g. entrance fees), limited resources (e.g., specific equipment) required for some types of physical activity, time constraints due to inflexible work schedules and family responsibilities, and cultural differences [8, 9, 10, 11, 12, 13, 14, 15, 16, 17]. Furthermore, literature shows that the neighborhood built environment in high-income countries is associated with physical activity among adults with low SES and that they are more often exposed to an unhealthy food environment [18, 19, 20]. Considering these health inequities across different SES groups, the role of the local environment (such as the physical, food and social environments) may be of greater importance in adopting a healthy lifestyle for lower SES groups and in disadvantaged neighborhoods [21].

Over the past decade, research exploring how aspects of physical activity and food environments may contribute to current obesity levels has significantly increased [14, 15, 22, 23 ,24, 25]. Physical activity and eating behaviors are complex and often interrelated. For example, low levels of physical activity among adults have been associated with disinhibition and cravings for savory foods, as well as an increase in weight after one-year follow-up [26]. Thus, people who eat healthier might have healthier physical activity patterns and vice versa. However, studies systematically examining the influence of the environment on both behaviors are limited [15, 27, 28]. Therefore, previous research has recommended to incorporate both physical activity and diet in the same study to better understand the complex association between the environment and obesity [29, 30].

Previously, studies were mostly conducted in Anglo-American countries (e.g. [22, 23, 31, 32, 33]) but in recent years, the number of studies in Europe has increased [13, 21, 34, 35, 36, 37, 38]. Most of these studies focus on either urban or rural environments, while studies focusing on peri-urban environments that have both urban and rural characteristics are scarce [39]. This goes beyond the fact that obesity is not just an urban or rural phenomenon and that 35.5% of the European population lives in peri-urban areas [40]. Additionally, the number of such areas is increasing faster than that of the traditional core cities [41]. Results from the Belgian Health Interview Survey in 2018 show that obesity is similar in urban, peri-urban and rural areas [42]. However, most research regarding physical activity or eating behavior in Belgium was conducted in urban centers. Since obesity prevalence is similar independently of the area and research systematically focused mostly on urban and (however less so in Europe) on rural environments, there is a need to investigate peri-urban areas as these are common living environments in Belgium in particular and in Europe in general [41].

Geographic information systems (GIS) are often employed to objectively record elements of the physical environment that are related to physical activity and diet [43, 44, 45]. However, several studies have criticized GIS for masking alternative versions of social reality [46] by excluding multiple perspectives, context and subjectivity by excluding non-cartographic spatial knowledge, such as people’s perceptions of places, and neglecting perspectives of minorities and disadvantaged groups [47]. The interwoven nature of person and place requires methods that can explore both objective and subjective relationships [48]. The reviews by Feng, Caspi, Mackenbach and Orstad point out that there are quite some inconsistencies in findings between studies focusing on objective versus perceived environments and the association with physical activity, healthy eating and obesity [24, 49, 50, 51]. For example, objectively measured high walkable areas that are perceived as poor walkable by residents are associated to decreased walking behavior in comparison with those where perceptions match the objective classification [52]. A systematic review by Orstad (2017) and colleagues argues that perceived neighborhood environment variables are significantly associated with physical activity at slightly higher rates than objective neighborhood environment variables [51]. They conclude that both objective and perceived measures of the neighborhood environment are related but distinct constructs that account for unique variance in physical activity, therefore it may be necessary to use both to examine the relationship between the built environment and physical activity [51, 53]. Another example is that a mismatch between non-reporting of an objectively measured close availability of a supermarket is significantly related to fewer fruits and vegetables consumption [54]. Caspi and colleagues (2012) argue that perceived measures of food environment may be more strongly related to dietary behavior than objective measures [54]. Citizen’s perception of their municipality might predict or mediate the relationship between physical activity, eating behavior and the objective environment [15, 51, 52]. However, studies combining both measures are still limited [15]. Furthermore, Roda et al. (2016) argued that better matches between perceived and objective measures of the obesogenic environment were observed in high-residential neighborhoods, they argue that future studies should focus on low(er)-residential neighborhoods, such as peri-urban or rural areas [55].

Community-based participatory research (CBPR) is a promising approach for health promotion in local communities [56]. It is a bottom-up approach engaging community members in participatory ways to identify mutual issues and take action to address them [57]. CBPR approaches have been effectively used to explore barriers and facilitators regarding physical activity and healthy eating [58], as well as to identify and implement interventions to promote a healthy lifestyle [56, 59, 60, 61, 62]. CBPR recognizes the importance of adaptation and effective translation of research findings to diverse settings and at multiple levels [63]. By including community members in identifying barriers and facilitators, it allows to integrate novel and community-sourced ideas for practical intervention planning with high external validity where community members are valuable contributing members of the process [64]. Furthermore, there is proof that empowerment can improve health among different subpopulations, especially those at risk for social exclusion, through knowledge exchange, strengthening self-efficacy, perceived social support etc. [34, 61, 64]. Empowerment, broadly defined as a process whereby individuals, communities or organizations experience more power and control over decisions or actions that influence their lives, can be enhanced through CBPR at both the individual and community level [65, 66]. It is increasingly seen as a goal of health promotion, whereby people experience more control over underlying health determinants, but also indirectly because of efforts to mobilize, organize and educate people [67].

Findings from aforementioned studies [15, 17, 38, 44, 50, 64] led to the design of the CIVISANO project to tackle inequities in health enhancing lifestyle within a municipality environment through a mixed-method approach. By integrating both objective and perceived measures, including CBPR approaches, CIVISANO aims to study the complex processes enacted between socioeconomically disadvantaged adults and their local environment in two peri-urban Flemish municipalities in Belgium and aims to identify possible context specific local actions for healthy lifestyle promotion. Due to the strong socioeconomic profile of overweight and obesity [42, 68, 69, 70] and the possibility of reaching these socioeconomically disadvantaged groups more effectively through environmental and community-based approaches than through individual interventions [56, 61, 64], the study will focus on the barriers and facilitators towards physical activity and healthy eating behavior within the local environment of socioeconomically disadvantaged adults. A multidimensional approach towards SES will be applied, because of the heterogeneity of socioeconomic disadvantages in the municipality context. The aim of this paper is to describe the protocol of the CIVISANO study.

## Design

### Objectives and study design

The overall goal of the CIVISANO project (2020–2023) is to identify both objective and perceived environmental factors (e.g. physical, sociocultural, economic, political) that facilitate or impede physical activity and healthy eating behavior among socioeconomically disadvantaged adults (25–65 years old) in two peri-urban municipalities in Flanders (Belgium). A mixed-method approach, using both quantitative and qualitative techniques, will be applied to obtain this objective. The mixed-method approach will reveal different aspects of the same environmental factors. Data on the ‘objective’ environment will be collected with quantitative methods. The qualitative methods will provide more in-depth and illustrative information to understand and contextualize the various dimensions in perceptions towards local food and physical activity environments that cannot be quantified [71]. GIS will be used to objectively capture the built environment, while a questionnaire and CBPR approaches will be used to gain insight into which components of the environment are perceived as barriers or facilitators to physical activity and healthy eating behavior by socioeconomically disadvantaged adults. These methods will be explained below in the Methods section (I-III). In addition, we aim to identify actions and/or interventions that can be taken to promote physical activity and healthy eating behavior in the local environment. Furthermore, we aim to evaluate the process of the CBPR approach through an individual community-related empowerment evaluation and group session evaluations based on CBPR principles. The process evaluation will be explained in the Methods section (IV). Each municipality will serve as a separate case study.

### Study area

Two medium-sized peri-urban municipalities, Duffel and Herselt, will be the setting for the CIVISANO project. Initially, 12 Flemish municipalities were considered as potential candidates based on distinct characteristics. The selection was based on geographic and linguistic area (Flemish region of Belgium, mainly Dutch-speaking, *n* = 300), population size (13,000–23,000 inhabitants, *n* = 93) [72] and socioeconomic indicators for municipalities (e.g. exclusion of industrial areas, extremely rural areas, richest municipalities, etc.) from the Belfius Index 2018 (*n* = 37). The Belfius Index 2018 studied and compared the sociodemographic typology of all municipalities in Belgium [73]. Lastly, feasibility of transport (distance and time) to the municipalities has been considered, as the Brussels-based researchers will have to travel back and forth a lot due to the community-based participatory approach (less than 2 hours travel time one-way, *n* = 12). Finally, two municipalities decided to participate.

Both municipalities are located in the province of Antwerp, in the Flanders region of Belgium. The municipality of Duffel is 22.6 km^2^ and counts 17,664 inhabitants, which accounts for an average density of 781.0 inhabitants/km^2^ [72]. The municipality of Herselt is 52.4 km^2^ and counts 14,521 inhabitants which means an average density of 277.0 inhabitants/km^2^ [72]. While Herselt contains multiple sub-municipalities, Duffel is a monocentric municipality.

### Study population

The study participants are socioeconomically disadvantaged adults between 25 and 65 years old who live in Duffel or Herselt. The lower age limit was chosen because it can be assumed that this age group is no longer in school and lives in a relatively stable housing situation, in contrast to a younger population that may live in student housing and is more likely to have family-related responsibilities (‘role-model’) [74, 75]. The higher age limit (65 years old) was chosen because this cut-off is the entitled retirement age in Belgium, indicating that we focus on the working population [76]. Furthermore, this cut-off is often used in research and policy to define older people [77]. Additionally, participants were required to be Dutch speaking to be able to complete the questionnaires and to participate in the individual interviews and focus group discussions. In 2019, the population between the ages of 20 and 65 in Duffel and Herselt consisted of 9248 residents and 7728 residents respectively [78].

The number of socioeconomically disadvantaged adults between 25 and 65 years old in the population is not recorded at the municipality level in Flanders. However, on a global population level in Flanders, it is known that 18.4% (adults between 25 and 65 years old) has a lower SES based on educational level (cut off low educational status = no higher educational degree) [79]. In the literature, SES is often based on a single-item indicator such as educational attainment or income level. Relying on a single indicator of SES does not account for short-term fluctuations that may affect individuals, such as unemployment. Using multiple indicators that can account for an individual’s SES more holistically has been shown to be a more reliable approach [80]. Therefore, respondents are classified as socioeconomically disadvantaged in this project if they meet one of the following criteria: have an income below the national minimum income level, have not obtained a higher educational degree or give themselves a score of lower than five on the MacArthur scale of social status [81, 82, 83, 84, 85, 86].

### Recruitment

As the project consists of several methods with different participants, the recruitment descriptions are divided into a quantitative part (questionnaire and GIS) and a qualitative (photovoice, walk-along, individual map creation and group model building) part.

#### Recruitment quantitative part

The questionnaire will be open to the entire population between the ages of 25 and 65 years old in both municipalities and invitations to participate will be distributed through local journals, social media, posters, and flyers in public places. In this study, an overrepresentation of socioeconomically disadvantaged adults is intended. Therefore, active recruitment of this group will be done through door-to-door visits in socioeconomically disadvantaged neighborhoods (e.g. pre-identified areas by the local authorities, areas with social housing, areas with lower private renting costs). Depending on the COVID-19 measures in place at the time of recruitment, a volunteer will either ring the doorbell and administer the questionnaire with a tablet or leave the paper questionnaire or a flyer (with web-link and QR-code to fill in the questionnaire online) in the mailbox, with a proposition to come back and pick it up a week later, or ask to drop the questionnaire in a predefined central mailbox in the municipality. Additionally, after a respondent has completed the questionnaire, a snowballing technique (meaning that participants refer potential participants from their social circle to researchers) will be applied. Furthermore, members of the research team will be present in public places, at local social organizations and at local events and food distributions. Similar methods have been proven to be effective to reach out to more disadvantaged and ‘hidden’ groups of people [87]. Local organizations that support socioeconomically disadvantaged adults, such as local service centers or local health care centers, will invite them to participate. Announcements will be made in the local journal and on the website of the municipality to increase visibility. Thus, apart from age (25–65 years old) and residence in the municipality, no specific inclusion criteria will apply. The criteria to determine if a participant has a low or high SES will only be checked after participation, as explained below in the questionnaire section. To counter potential stigmatization, the researchers and volunteers who spread the questionnaires will not emphasize ‘socioeconomic disadvantage’, nor is it written in the survey or informed consent, as the survey is available to all residents of the municipality. The language used during recruitment is carefully vetted to be people-first and as neutral as possible. No incentive for participation in the quantitative part will be provided for the participants.

#### Recruitment qualitative part

In the questionnaire, a section will be added where participants can express their interest to join the participatory workshops that will be organized later in the project. Furthermore, the door-to-door visits and recruitment at public places, at local social organizations, and during local events and food distributions as described above will also be performed. Furthermore, participants will be asked at the end of each session if they know other persons that might be interested to join (cfr. Snowball technique). Before participation, eligibility will be checked. The inclusion criteria are age (25–65 years old), being a resident in the municipality and having one of the following indicators: no higher educational degree [88], no current paid or very low paid work [89], current perceived economic difficulties [90] and a perceived SES of 5 or less on a 10-point scale (where 10 and 1 represent the highest and lowest perceived SES, respectively) [85]. An incentive - a voucher of 10€ to be used in local shops in the municipalities - will be provided to participants who attend the participatory workshops.

## Methods

In each municipality, a questionnaire will be administered among all residents independent of SES, which will serve as the basis for the geographic analysis. The geographic analysis will study the association between the physical activity and food environments and the dietary habits and physical activity behavior of participants according to their socioeconomic status. Walk-along, photovoice and individual map creation methods will be conducted afterwards among socioeconomically disadvantaged residents, followed by group model building sessions. These methods are part of the CBPR approach and will be explained in detail below under methods section III (i-iii).

Furthermore, the CBPR approach includes partnership building, experience-sharing, capacity building, empowerment and co-learning among partners [64, 91]. Therefore, the multidisciplinary research group, a local steering group (a local policy officer, a local staff member of the local OCMW (public center for social welfare) and a staff member of the regional health prevention organization), a broader stakeholder group (local representatives of public organizations in the municipality such as sports infrastructures, food stores, physiotherapists, dieticians, social workers, etc.) and socioeconomically disadvantaged citizens living in the municipality will work together to explore the local determinants of a healthy lifestyle and search for possible local (policy) actions in each municipality. Meetings with the multidisciplinary research group and with the local steering group in each municipality will take place every month, while meetings with the stakeholder group will take bi-annually. Establishing monthly meetings with the local steering group will allow for effective and rapid communication and is important to harbor the quality of the study in unforeseen circumstances (e.g. the COVID-19 pandemic).

**Fig. 1:**
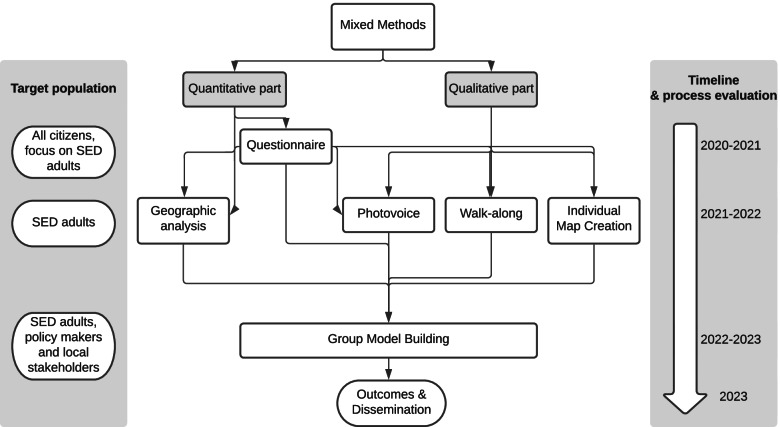
overview and timeline of methods of the CIVISANO study

A process evaluation will be conducted, consisting of an empowerment evaluation at the beginning and at the end of the project and session evaluations at the end of each focus group session and stakeholder meeting. Results will be disseminated throughout and at the end of the project, as will be argued in the discussion section. An overview of the methods that will be used in the CIVISANO study can be found in Fig. 1.

### I. Questionnaire

To gain information on individual sociodemographic characteristics, health behavior and perceptions towards the local environment, adult residents from both municipalities will be invited to complete a questionnaire. Multiple members of the same household are allowed to complete the questionnaire since they can have different obesity-related behaviors.

The aimed sample size is a total of 254 participants, consisting of 127 socioeconomically disadvantaged and 127 non-disadvantaged participants. The calculation was performed to support the primary research questions in the quantitative part of the study i.e. the examine the association between either the food environment and dietary habits according to socioeconomic status and to examine the association between physical activity environment and physical activity according to socioeconomic status. For this purpose, a linear regression analysis will be conducted. The outcome variables were the frequency of dietary habits (e.g. fruit- and vegetable, sugar-sweetened beverages and fast-food consumption) and the frequency of domain-specific physical activity with 12 predictors (age, gender, SES, living situation, BMI, subjective health status, food insecurity, transport, perceptions of accessibility and availability of the food−/physical activity environment, density and proximity of food outlets/green spaces) and a medium effect size of 0.15.

The questionnaire employs both a paper-and-pencil interview approach (PAPI) and a computer assisted web interview (CAWI) mode. Both the CAWI and PAPI mode of the questionnaire are self-administered and contain the same questions. The questionnaire includes the following items: sociodemographic information, subjective SES, subjective health and anthropometrics, dietary habits, food security, physical activity, and perceptions on the food-, physical activity-, and social environment [92]. Through multiple discussions with the research group, a selection of variables has been chosen based on their relation with physical activity, eating behavior and the local environment. Most of these variables were derived from the Local Health Interview Survey (Local HIS) of 2019 [92]. Variables included in the Local HIS 2019 were on their turn derived from the Belgian National Health Interview Survey of 2018 [93]. However, the selection for the Local HIS was based on extensive literature review to identify the variables which best describe the health-related issues that are monitored on a local administrative level instead of a regional or national one.

Due to the scope of this project, extra variables in addition to those selected from the Local HIS were added to the questionnaire. To assess factors related to the physical activity and food environments that could influence physical activity and healthy eating behavior, items from the Flemish version of the sustainable prevention of obesity through integrated strategies project (SPOTLIGHT) and the perceived nutrition environments measures survey (NEMS-P) were included [38, 94]. To measure social capital on the neighborhood level, questions on the social environment were added [95]. In addition, questions assessing food security during the last 3 months from the current population survey food security supplement (CPS-FSS) were included as well [96]. Discussions with members of the steering committees in the municipalities indicated that questions on motivators and deterrents for physical activity as well as membership of sports clubs were lacking in the questionnaire. As such, questions assessing those factors which have been used in previous studies on physical activity have also been included [97, 98]. This yielded a survey of 40 questions which can be completed in 15 to 20 minutes. The questionnaire has been designed using LimeSurvey 5.3.26 by applying a simple layout with features that make it easy to fill in for participants (e.g. short items and Likert scales) which has been shown to improve participants response rate [99, 100] . Before completion, the questionnaire has been read and redacted by Wablieft,^1^ a Flemish organization that advocates accessibility and comprehensibility of the Dutch language for disadvantaged groups.

### II. Geographic analysis

The geographic analysis will localize each respondent of the questionnaire by adding a geographic component to the questionnaire data, identify and compute relevant objective indicators characterizing each respondent’s localization in terms of the objective food and physical activity environments using ArcGIS Pro 2.8.7 geographic information systems (GIS) software. Furthermore, a participatory GIS workshop will take place to map the commuting behavior of respondents from socioeconomically disadvantaged groups.

#### i. Residential locations

To ensure that the privacy of respondents is protected when the detailed maps are published, the home address of survey respondents will not be used. Instead, when completing the survey participants will be asked to indicate the intersection closest to their home address on a map indicating all intersections in their municipality. Afterwards, the intersection closest to their home address will be geocoded as latitude and longitude values and used as a proxy for the respondent’s residential address to link the questionnaire data with spatial data from other sources. To enable the localization of each respondent, both the PAPI and CAWI mode of the questionnaire include a localization question.

#### ii. Objective indicators on food environments

The association between the Retail Food Environment, which consists of the ‘community retail food environment’ (the local opportunities to access food) and the ‘consumer retail food environment’ (the environment within and around food outlets) [101], and obesity has generated considerable attention in research during the last decade [102]. Within this study both the community and consumer retail food environments will be assessed. The community food environment which encompasses the number, type, location, and accessibility of food outlets will be objectively assessed by analyzing data from the Locatus dataset, a commercial dataset containing information on the types and locations of all food retailers in Belgium. This dataset has previously been validated against field audit data in the Netherlands and showed good to excellent agreement statistics [103]. From the Locatus dataset, a map of all primary food outlets (address points) in the municipalities will be created. These are food outlets that sell food as a primary activity (e.g. supermarkets, butchers, restaurants etc.) in contrast to outlets that sell food as a secondary activity (e.g. sport centers, cinema’s, game halls etc.). Food outlets will be divided into the following categories: fast food restaurants (including chains and locally owned restaurants, food delivery and takeaway outlets), full service restaurants, supermarkets, local food shops primarily selling vegetables such as greengrocers, local food shops primarily selling animal products (e.g. butchers, fish mongers etc.), local food shops primarily selling bread such as bakeries, local food shops primarily selling organic products (e.g. organic shops, farm shops etc.), confectionery stores and convenience stores (Vandevijvere & Smets, 2021, unpublished data). To assess the consumer food environment, in-store audits of the availability of healthy and unhealthy products according to Flemish dietary guidelines [104] and their strategic placement inside the store will be conducted in a sample of supermarkets and convenience stores in both municipalities.

For each survey participant, indicators of the community and consumer food environment will be calculated on the individual level by employing network buffers of 500 m and 1000 m around each intersection closest to the respondent’s address. The 500 m and 1000 m buffers were chosen based on previous studies conducted internationally and in Belgium as part of the ‘International Physical Activity and Environment Network’ (IPEN) which recommends the use of street network buffers of 500 m and 1000 m around participants’ residences to develop a standardized spatial definition of a ‘neighborhood’ that can be used to compare results between countries [105]. The following indicators will be calculated:

Community food environment:

- Food accessibility will be measured through the density of food outlets (see categories stated above), corrected for population density per street-network buffer of 500 m and 1000 m around participants closest intersection and on the municipal level. The food outlets are scored based on the healthiness of the available products. These scores range from 1 (very unhealthy) to 5 (very healthy) and have been developed based on an expert opinion study conducted in Belgium (Vandevijvere, 2021, unpublished data).
- Food proximity will be defined as the distance between the intersection and the closest food outlet of each type. It will be measured by travel distance using the street network within street-network buffers of 500 m and 1000 m .

Consumer food environment:

- Food availability, prominence and promotion within the consumer food environment (e.g. a selection of supermarkets and convenience stores in each municipality) will be measured through evaluating the availability of healthy (e.g. fresh fruit and vegetables, frozen fruit and vegetables) versus unhealthy food groups (e.g. confectionary, crisps/chips, soft drink and sweet biscuits) during field-audits in a representative sample of food outlets in the municipalities. The following indicators will be assessed:
  - ▪ Ratio of cumulative linear shelf length for healthy versus unhealthy foods
  - ▪ Number and proportion of junk-food-free checkouts
  - ▪ Number and proportion of junk-food-free end-of-aisle endcaps (front and back)
  - ▪ Number and proportion of junk-food-free promotions in store

These indicators are based on INFORMAS recommendations [106]. A previous validation study showed that they can be used as a valid indicator of the relative availability of healthy versus unhealthy foods [107].

#### iii. Objective indicators of the physical activity environment

The physical activity environment is a subset of the broadly defined ‘physical environment,’ which includes elements of built and natural environments [108]. The built environment refers to all buildings, spaces and objects that are created and modified by humans. It includes homes, schools, workplaces, recreational facilities, and infrastructure [108]. Natural environments include open spaces in and around towns and cities, including parks, nature areas, canals, the coast and beaches and the countryside (e.g. farmland, woodland, hills, and rivers) [109]. In this study, the physical activity environment will encompass features of the built environment as well as features of the natural environment.

Buildings will be described in terms of residences/habitation, workplaces, schools, daycare facilities, food retailers (e.g. supermarkets, convenience stores, restaurants, fast food chains etc.) and sports facilities (e.g. sport halls, gyms, pools etc.). The network infrastructure will be described by the street network supporting daily mobility. Data on the buildings and network infrastructure in the municipalities is made available by the Flemish Government within the ‘Grootschalig referentiebestand’(GRB) (Large-scale reference database of Flanders), which contains – among others – spatial data on buildings, roads, rail- and waterways [110]. Data supporting physical activity such as sport facilities, swimming pools etc. will be obtained through the Locatus dataset. Data on the overall availability of green spaces will be derived from the ‘Groenkaart Vlaanderen’ [111]. While data on public green spaces will be derived from the Corine Land Cover dataset, a European land use dataset developed by the European Environment Agency [112].

Based on the mentioned data sources, the following indicators will be computed using the intersection closest to the residential address, to characterize each respondent’s physical activity environment:

- Availability of green spaces will be described by the percentage of surface area dedicated to green space per network buffer of 500 m and 1000 m around each intersection and within each municipality.
- Accessibility of green spaces will be described by the average distance per residential location from the closest intersection to the closest public green space along the street network.
- Availability of sports facilities will be described by the count of facilities within 500 m and 1000 m network buffers around each intersection.
- Accessibility of sports facilities will be described by the average distance per residential location from the closest intersection to the closest sports facility along the street network.
- Walkability will be calculated based on previously developed methods. A walkability index was created for the entire region of Flanders by the Department of Environment and Spatial Planning together with the Flemish Institute of Healthy Living [113], which will be utilized by applying network buffers of 500 m and 1000 m around each intersection. The Flemish walkability index uses land use data, population density based on the census and road network data to calculate the following variables: street connectivity, net residential density, and land use mix. Since the walkability score relies solely on objectively measured macro-features and does not include the perceptions of community members on the built environment and because the index has been developed for urban areas, peri-urban or rural areas tend to score poorly for walkability. Therefore, the walkability score will be supplemented with (qualitative) data collected during the participatory GIS part of this study, as well as with micro-scale features (e.g. presence of sidewalks, trees, crossing signals, street lightning) of the built environment.

#### iv. Participatory GIS workshop

To map the commuting mobility behavior of participants from socioeconomically disadvantaged groups, a participatory GIS approach will be used. Within the CIVISANO project, this approach will be referred to as Individual Map Creation (IMC). IMC will focus on the construction of digital maps by the respondents, depicting the routes that they regularly take for commuting purposes. The routes will be digitized using an online platform which has been developed by the Network for Sustainable Mobility Research from Ghent University for the FietsSTEM for schools’ project^2^ (Ghent University, n.d.). In the online environment, participants will indicate their starting point and the route they take for commuting to a range of destinations. Routes will be split up into multiple sections and for each section, participants will be able to indicate which type of transportation they use. Furthermore, participants will be able to voice their opinion on each section of the route such as to indicate that a particular intersection makes them feel unpleasant or unsafe. A one-to-one approach between the researcher and a participant will be used for the mapping. During the construction of the routes, a semi-structured interview will take place. This interview will employ an interview guide based on previous research by Kegler et al. (2015) and will include open-ended questions focusing on participants perceptions of their environment and will include questions on themes such as safety (e.g. heavy traffic) and aesthetics (e.g. quality of walking- and cycle paths) [114].

### III. Community-based participatory research methods

Photovoice, walk-along and group model building techniques will be used as community-based participatory tools to document and actively explore the perceived environmental factors stimulating or impeding physical activity and healthy eating behavior by asking open questions, listening and observing [115]. These low-threshold ways of interaction to reach and involve socioeconomically disadvantaged groups have the potential to reduce health inequities by increasing the saliency of findings and proposed actions, and through empowerment processes [34, 61].

The environmental factors and actions stimulating or impeding physical activity and healthy eating behavior will be captured through the ANalysis Grid for Environments Linked to Obesity or ANGELO framework. It is a tool based on the ecological model for understanding obesity and developed by Swinburn et al. (1999) to identify and categorize various obesogenic components in the environment. Environmental elements can be considered “obesogenic” (advancing weight gain or barriers for adapting healthy behaviors) or “leptogenic” (promoting weight loss or enhancers to adapt healthy behaviors) in relation to maintaining a healthy body weight [116]. A 2 × 4 grid dissects the obesogenic environments into two environmental sizes (micro-environments called settings, and macro-environments called sectors) and into four environmental types, namely physical (what is available), economic (what are the costs), policy (what are the rules) and sociocultural (what are the attitudes, beliefs, and values) environment [116].

#### i. Photovoice

In both municipalities, the photovoice method will be used to explore the role of environmental factors on dietary behavior among socioeconomically disadvantaged adults. Participants may be the same as those who participate to the walk-along interviews, but new participants can be invited as well. The total number of participants in the photovoice will depend on when an overall saturation of information is achieved [117]. The number of participants for each focus group will be maximum 10.

The photovoice method will be used to capture the participants’ perception of their food environment. It is a methodology that uses photographs as a way for participants to share a story and to engage them in the data-collection process [118]. Participants take photographs of anything in their environment that influences their eating behavior for 2 weeks [119, 120]. Disposable or refurbished digital cameras will be available for those who do not have a smartphone. They will be asked to write in a logbook their thoughts on why they took a particular photograph [119]. Three meetings will take place: one to explain the project and provide a photovoice training, a second to discuss the taken photographs and to identify the environmental factors that facilitate or impede their eating behavior and to organize them within the ANGELO framework, and a third one to identify possible (policy) actions. Deductive and inductive analysis of the determinants and actions will be organized within the ANGELO framework. The coding and organization of determinants within the framework will be started by the participants during the workshops and continued by the researchers during the different meetings. During each session, participants can check the statements previously made and can make adaptations (= member checking). This feedback loop provides a participative contribution to the data analysis [121].

Photovoice is an effective community-based participatory research tool for advancing health equity [118, 122]. It enables participants to identify, define and enhance certain situations within their community according to their specific concerns and priorities by the creation and evaluation of photographs [25, 118, 121]. The use of the visual image accompanied by the personal stories has a distinctive capacity to engage hard-to-reach groups and to enable the participants to reflect on, verbalize, and share their experiences, expertise and knowledge to gain an in-depth understanding of their perceived factors affecting healthy eating behavior [25, 118, 121, 123, 124]. By its capacity to engage hard-to-reach groups and to elicit open and honest conversations, photovoice appears to contribute to an enhanced understanding of community assets and needs [121].

#### ii. Walk-along

In both municipalities, the walk-along interviews will take place to explore the role of local environmental factors on recreational walking behavior among socioeconomically disadvantaged adults. Participants may be the same as those participating in the photovoice workshops, but new participants can be invited as well. The total number of participants for the walk-along will depend on when an overall saturation of information is achieved [117]. The maximum number of participants for each focus group will be 10.

During the walk-along, the researcher interviews the participant while walking in his/her local environment. By asking open questions, listening and observing, the researcher actively explores the participants’ subjective experiences regarding their physical activity, walking in particular, as they move through and interact with their local environment [115, 125]. It is therefore a combination of two more traditional qualitative methods: field observation and in-depth interviews, which enables to observe participants’ spatial practices, while accessing their experiences and interpretations of their local residential context at the same time [115, 125]. One individual walk per participant (accompanied by a researcher) will take place, followed by a focus group where experiences and findings from the individual walks will be shared and discussed which will also serve as member checking. Environmental factors that facilitate or impede physical activity, will be organized within the ANGELO framework. During this focus group potential actions will be discussed within the same framework as well. Deductive and inductive analysis of the determinants and actions will be organized within ANGELO framework. The coding and organization of determinants within the framework will be started by the participants during the workshops and will be continued by the researchers during the different meetings. During each session, participants can check the statements previously made and can make adaptations. This feedback loop is called ‘member checking’ and provides a participative contribution to the data analysis [117, 121].

#### iii. Group model building (GMB)

The group model building technique will be used in each municipality with both members of the stakeholder group and socioeconomically disadvantaged adults (only if they have already participated in the walk-along or photovoice workshops) to explore the perceived system drivers behind physical activity and healthy eating behavior and to identify possible (local) actions based on the findings [126, 127, 128]. To understand how different elements of a system interact and relate through multiple feedback loops to produce the behavior of concern, causal loop diagrams (CLD) will be developed, based on the identified determinants during the qualitative workshops and objective analysis [17, 126, 127, 128]. The CLD’s will provide shared insights and new perspectives on the perceived role of the local environment on physical activity and healthy eating behavior. These insights will be used for further dialogue on local actions or intervention development to address the system drivers of the local obesogenic environment among socioeconomically disadvantaged groups from different perspectives [127, 129]. This method has been proven to be highly effective for engaging communities in identifying different determinants that play a role in developing a healthy municipality, developing ‘bottom-up’ solutions and exploring barriers to action [128, 130]. The GMB workshops will be tackling ‘obesogenic environments’, so there will be a focus on both physical activity and food environments at the same time. The group will consist of 10–15 participants of the stakeholder group and socioeconomically disadvantaged adults. Three workshops will take place: one to scope the central issue (environmental factors influencing physical activity and healthy eating behavior among socioeconomically disadvantaged groups) and to make sure that there is a shared understanding (creation of a preliminary CLD), a second to find the (inter)relation of variables related to the central issue (creation of a CLD), and a third and final to identify and prioritize possible interventions.

### IV. Process evaluation

In this process evaluation, we will focus on how the individual community-related empowerment evolves during the project and how participants experience the workshops.

#### i. Individual community-related empowerment (ICRE)

The scope of this study will be at the level of the individual community-related empowerment (ICRE). In literature on community health, empowerment is often viewed as a process starting with the individual and transferring over into the development of small mutual groups, community organizations, partnerships, and eventually leading to community empowerment, social change and political actions [66, 67]. Several researchers argue that individual community-related empowerment is a prerequisite for community empowerment and social change [66]. A universal measure of empowerment does not exist and is difficult and even undesirable to develop, as it differs among individuals, cultures and contexts and it may fluctuate over time [66, 131]. Furthermore, empowerment is a long and slow process, implicating that an empowerment evaluation in a project context is more an evaluation of changes in the process rather than a particular outcome [67].

The ICRE assessment that will be used in this study is developed by Kasmel & Tanggaard (2011) and based on the Mobilization Scale from Jakes & Shannon (2002) [66, 132]. It is a self-administered questionnaire with 18 items rated on a Likert 5-point scale (1 = ‘strongly disagree’ to 5 = ‘strongly agree’). It will assess community members’ (here stakeholders and socioeconomically disadvantaged adults) ratings of dimensions of ICRE before, during and after the workshops. ICRE is defined by combining multiple components, such as self-efficacy (or self-confidence), participation (or involvement in collective action), motivation to be involved in community action, intention or willingness to act in the public domain and critical awareness that community issues are serious [66]. The questionnaire will be proof-read by a community member to check for clarity and understanding.

#### ii. Session evaluation

When using participatory research methods, it is important to keep a reflective attitude, being aware of group and power dynamics and the influence of your position as academic researcher [133]. Therefore, each photovoice, walk-along or group model building session with participants and stakeholders, as well as the stakeholder meetings, will be evaluated through a short questionnaire. At the end of the project a focus group will take place with a random sample of participants and stakeholders where good practices, issues and room for improvement will be discussed. Through successive meetings with the same researchers, a bond of trust and an open space to share thoughts and experiences can be created which might make it easier to be honest about experiences.

The short questionnaire will be based on co-creation principles and will consist out of 14 statements that will be scored on a 5-point Likert scale (1 = ‘strongly disagree’ to 5 = ‘strongly agree’). The statements are the following: openness for new ideas and opinions, exchange of useful information, equal level of involvement, a climate of trust and openness, useful conversations, positive atmosphere, development of new insights, good feeling, clear common mission, equal influence on decision-making, respectful interactions, satisfaction with course and progress, use of comprehensible language, interesting and fascinating topics [134, 135]. Furthermore, through two additional open questions developed by the research group, the participants will be asked which elements facilitated or hindered discussions during the sessions. The researcher will also complete the same questionnaire and make reflection notes on perceived involvement and group dynamics.

##### Ethical clearance

This study is approved by the Medical Ethics Committee of the University Hospital Ghent (BC-09260) and will be carried out in line with the recommendations of the Belgian Data Protection Authority. All participants will have to sign an informed consent before participation.

## Discussion

Through the CIVISANO project, we aim to identify objective and perceived local environmental factors that play a role in the physical activity and nutrition behavior among socioeconomically disadvantaged adults in the understudied area of peri-urban municipalities. Furthermore, we hope to promote healthy behavior in the long-term through actions that can be installed after the project based on the identified determinants and proposed actions.

The project has been set up by a multidisciplinary team and integrates mixed-methods and community-based participatory approaches to purposefully impact on policy and practice towards a healthy lifestyle in the municipalities. Following recommendations from participatory research, all results will be disseminated within the municipalities and with the broader community throughout and at the end of the project [136, 137]. The ways of dissemination will be discussed with the participants. The project aims to provide the essential foundation for the municipality to develop actions in collaboration with socioeconomically disadvantaged residents to enhance physical activity and promote healthy eating for all.

A strength of this study is the focus on socioeconomically disadvantaged adults, whereby an multidimensional approach towards these socioeconomic characteristics is applied. As discussed in the introduction, low SES has most often been defined in research designs by educational level. However, this indicator will not encompass the entire effect of SES [138, 139, 140]. By applying a broad definition of SES instead of solely focusing on education a higher percentage of socioeconomically disadvantaged adults might be included into the project. The project outcomes and created models will focus on multiple mutually constituting SES characteristics, instead of treating it as a modifier [141, 142]. This may influence results since some environmental determinants are found solely to be related to obesity in specific subgroups [141]. Furthermore, interventions towards obesity such as educational, behavioral and pharmaceutical approaches only have a limited impact in socioeconomically disadvantaged adults [143, 144]. Previous research using a whole system approach or specifically targeting vulnerable groups have been promising to promote physical activity and healthy eating [126, 129, 144, 145].

Additionally, a strength of the project is the focus on the active recruitment (e.g. door-to-door visits, presence at local social organizations, events and food distributions) of socioeconomically disadvantaged groups, since it is known that these groups are considered as hard-to-reach and hard-to-engage in research (e.g. because of less access to internet). Especially for the survey, active recruitment may aid inclusion of socioeconomically disadvantaged groups compared to a solely online based approach [146]. Also during the CBPR methods, participants will be actively approached and involved in multiple stages of the project (e.g. identification environmental determinants and actions, group discussions with stakeholders and local policymakers, distribution of results). Furthermore, we wanted to use varied and multiple methods to evaluate which methods might work better with this specific population. The walk-along method will study the role of the local environment on recreational walking, while individual map creation will focus on the role of the local environment on walking as functional transport (e.g. walking to a shop). Walk-along interviews take place in a natural context and might enhance discussion which seemed suitable to discuss the role of the environment on recreational walking. The individual map creation, which is a participatory mapping approach, seemed more suitable to study walking for transport because it combines semi-structured interviews with the geographic information systems to analyze features related to specific routes in relation to findings from the questionnaire and the in-store audits. Photovoice will be used to study the role of the local food environment on eating behavior. The use of photographs makes it possible to capture a broad spectrum of environmental factors, which will not be captures through a walk-along interview as this implies the choice of a specific environment (e.g. walking interview in a food store or a walking interview to a specific store).

There are relatively few CBPR studies in the field of public health and obesity. This may be because not all studies using CBPR approaches include all elements of CBPR [57], which is often not possible due to lack of time or resources [147]. This is also the case in this study due to lack of time, budget, and because of a time delay due to COVID-19 (e.g. we cannot speak of a completely equal partnership between academics, the municipality and the socioeconomically disadvantaged adults). However, this is by our knowledge, the first study including CBPR approaches to tackle obesity-related lifestyles for adults in Belgium.

Another strength of this study is the combination of objective measures with perceptions to provide a robust assessment of the municipal environment. The closest intersection will be utilized as a proxy of the home addresses of the participants, which increases precision in comparison with the common use of statistical sectors (i.e. smallest administrative unit in Belgium) in research, while not compromising the privacy of participants. Furthermore, we expect to identify potential mismatches between the objective measured environment and the perception towards their environment among socioeconomically disadvantaged adults, which would correspond with earlier research done in this field [55, 148]. However, by adding qualitative data to the geographic analysis and to the questionnaire data, we will be able to provide more in-depth and illustrative information in order to understand the various dimensions of perceptions towards the local environment that cannot be quantified [13].

Limitations of this study include the cross-sectional study design which does not permit causal inferences and the lack of generalizability of the results due to non-random purposive sampling [149]. The sample is limited to residents of two Flemish peri-urban municipalities, therefore transferability may be limited as the environmental factors impacting physical activity and healthy eating will be mainly linked to the local context [39]. Research in other locations, can provide a more complete understanding of a broader range of environmental factors influencing physical activity and eating behavior among a wide sample of socioeconomically disadvantaged adults. Furthermore, conducting a questionnaire always contains the risk of biases as it is self-reported data. It may not reflect actual contexts, processes and outcomes. Adding qualitative data may diminish the risk, however not the same number of topics are tackled during the interviews and focus groups. Social desirability bias is a risk in both quantitative surveys and during interviews. However, to minimize socially desirable answers there are multiple formats (PAPI and CAWI) available to complete the questionnaires. During the participatory methods, answering questions takes place in a more spontaneous and natural way by engaging with the environment through photos or a walk, which might lower the social desirability bias. Additionally, focus group discussions might diminish the power relationship between researcher and participant, due to the dynamism between multiple participants and the position of the researcher who stands alone at that time. Additionally, the preparation and execution of this study takes place during the COVID-19 pandemic, which complicates relationship building among all partners and the active participatory approach towards socioeconomically disadvantaged adults. Furthermore, other studies have shown that a lot of people have negatively changed their physical activity and eating behavior during the COVID-19 pandemic [150, 151]. As the focus of this study is on the current situation, we will not be able to capture these differences. Furthermore, the duration of the project is too short to capture all project outcomes and the mid- and long-term impact, such as the initiation of local actions, influences on policy, health impact, etc. However, this project should be considered as a ‘preparatory phase’ for more specific community interventions. It will create a ground for possible change by giving voice to disadvantaged groups, by creating partnerships and enhancing community capacity and participation.

## Data Availability

The data that will support the findings of this study will be available from Sciensano but restrictions apply to the availability of these data, which were used under license for the current study, and so will not be publicly available. However data will be available from the authors upon reasonable request and with permission of Sciensano.

## Abbreviations

ANGELO: Analysis grid for environments linked to obesity
BMI: Body mass index
CAWI: Computer-assisted web interviewing
CBPR: Community-based participatory research
CLD: Causal loop diagram
GIS: Geographic information system
GMB: Group model building
ICRE: Individual community-related empowerment
IMC: Individual map creation
PAPI: Paper-and-pencil interview
SED: Socioeconomically disadvantaged
SES: Socioeconomic status

## Footnotes

1 http://www.wablieft.be/nl.

2 https://fietsbarometer.ugent.be/home.
